# Transcriptome analysis reveals the role of jasmonate in regulating maize spikelet opening and seed set under high temperature stress

**DOI:** 10.3389/fpls.2026.1710459

**Published:** 2026-03-04

**Authors:** Ling Guan, Yang Chen

**Affiliations:** 1 Biotechnology Research Institute, Chongqing Academy of Agricultural Sciences, Chongqing, China; 2 Biotechnology Research Institute, Chongqing Key Laboratory of Adversity Agriculture Research, Chongqing, China; 3 Key Laboratory of Evaluation and Utilization for Special Crops Germplasm Resource in the Southwest Mountains, Ministry of Agriculture and Rural Affairs, Chongqing, China

**Keywords:** jasmonic acid, maize, spikelet, transcription factors, transcriptome

## Abstract

**Introduction:**

Maize is a crucial cereal crop, yet it is highly susceptible to heat stress, which considerably limits its grain yield. The opening of spikelets is a critical prerequisite for pollen shedding. Jasmonate (JA) plays significant roles in responding to abiotic stress and regulating spikelet development in plants.

**Methods:**

To investigate the molecular mechanisms underlying heat tolerance in maize, a heat-tolerant inbred line, Chang 7-2 (C7), and two heat-sensitive inbred lines, Yu727 and Y8201 (Y7 and Y8), were exposed to heat stress, followed by JA application, and subsequently analyzed using RNA sequencing.

**Results and Discussion:**

Our results indicate that under heat stress conditions, JA markedly enhances the seed-setting rate, spikelet opening rate, and spikelet opening angle in both Y7 and Y8. Moreover, JA effectively alleviates oxidative stress induced by heat stress in maize. KEGG analysis identified phenylpropanoid biosynthesis, flavonoid biosynthesis, and starch and sucrose metabolism as potential contributors to JA-mediated heat stress resistance in maize. Finally, Venn analysis of differentially expressed genes (DEGs) identified three transcription factors (TFs) involved in JA-mediated heat stress resistance in maize: MYBS3 and WRKY33, which play positive roles, and HOX22, which plays a negative role. Our findings collectively elucidate a fundamental regulatory network mediated by JA that enhances maize yield under heat stress conditions, offering viable gene targets for the genetic enhancement of maize yield in such environments.

## Introduction

In the context of climate change, the global increase in the frequency and intensity of heat stress has severely impacted crop production and food security (Zhao et al., 2017; Magaña Ugarte et al., 2019). Rising temperatures and the prevalence of heatwaves are projected to diminish the yield of staple crops (Tigchelaar et al., 2018). High-temperature (HT) stress exerts additional adverse effects on maize (*Zea mays* L.) growth and development, manifesting as reduced plant stature, decreased pollen viability, and impaired seed set (Li and Howell, 2021; Wang et al., 2020b).

Maize, a critical crop for food, fuel, and feed, is extensively cultivated worldwide and is susceptible to various abiotic stress, especially HT stress (Yang et al., 2023; Hou et al., 2025). During HT stress, the seed setting rate of maize is significantly constrained, leading to substantial reductions in grain yield (Borrás and Vitantonio-Mazzini, 2018; Deryng et al., 2014). Furthermore, the seed set in maize is intricately linked to various growth processes, including the differentiation and development of male and female organs, tassel and pollen shedding, silk emergence, the synchrony of pollen shedding and silking, and pollen germination and pollen tube elongation (Dresselhaus and Franklin-Tong, 2013). Each of these processes exhibits sensitivity to HT stress, thereby complicating the investigation of HT-induced yield loss in maize. Previous research focusing on short episodes of heat stress around anthesis in crops such as maize, rice (*Oryza sativa* L.), wheat (*Triticum aestivum* L.), and peanut (*Arachis hypogaea* L.) has identified sporogenesis, fertilization, and subsequent embryo formation as the most sensitive stages, resulting in significant kernel sterility (Shi et al., 2015; Teng et al., 2025; Prasad and Djanaguiraman, 2014; Vara Prasad et al., 2001). It has been established that the optimal temperature for maize anthesis is 30.5°C (Sánchez et al., 2014). Temperatures exceeding 38°C can impede maize pollen germination, leading to a reduction in pollen quantity due to inhibited panicle emergence (Sánchez et al., 2014; Prasad et al., 2006, Wang et al., 2020a). Although the anthesis stage has been demonstrated to be highly sensitive to heat stress, the effects of heat stress during maize anthesis are infrequently addressed. Spikelet opening is a critical prerequisite for pollen shedding and is succeeded by anther emergence from the floret, anther dehiscence, and pollen release from the pores at the anther tip, all of which are sensitive to ambient temperature (Keijzer et al., 1996, Dafni et al., 2000, Chen et al., 2020). Impairment of spikelet opening due to heat stress poses a significant threat to seed set in maize, given the considerable distance between the tassel and the ear (Chen et al., 2020). Enhancing spikelet opening is anticipated to improve plant tolerance to elevated temperatures and heatwaves. However, the morphophysiological and molecular mechanisms underlying spikelet opening in response to HT stress in maize remain inadequately understood.

It is widely recognized that phytohormones function as signaling molecules, playing crucial roles not only in regulating plant growth and development but also in mediating physiological responses to various biotic and abiotic stresses, including HT stress (Turner et al., 2002, Trang Nguyen et al., 2019; Wani et al., 2016). Jasmonates, encompassing jasmonic acid and its derivatives, are pivotal in responding to biotic and abiotic stresses and have emerged as a significant class of growth regulators, influencing flower development, fertility, stamen development, and sex determination (Yuan and Zhang, 2015; Acosta and Przybyl, 2019). In rice, JA have been demonstrated to regulate spikelet development (Kim et al., 2009). For instance, the JA content in the inflorescences and spikelets of the *eg1–3* mutant is reduced, and exogenous JA application can partially restore its spikelet development (Cai et al., 2014). Furthermore, the *EG1* gene plays a role in sustaining floret development under HT stress (Zhang et al., 2016).

Plants have evolved a multitude of physiological, molecular, and metabolic mechanisms to perceive and transduce environmental signals (Saini et al., 2022; Haider et al., 2021). Under HT stress, the generation of reactive oxygen species (ROS) within the plant body induces varying degrees of oxidative stress (Ruan et al., 2024). Consequently, there is an increase in the enzymatic activities of catalase (CAT) and peroxidase (POD), as well as in the levels of malondialdehyde (MDA) and proline (Pro) (Mittler et al., 2012, Guo et al., 2024). Furthermore, heat stress triggers the TF-mediated expression of various genes in plants, including those encoding heat shock factors (HSF), basic leucine zipper (bZIP), NAC, MYB, and WRKY (Cheng et al., 2021; Scharf et al., 2012; Ruan et al., 2024; El-Sappah et al., 2022). For instance, the overexpression of *ZmWRKY106* has been shown to enhance drought and heat tolerance in transgenic Arabidopsis by modulating stress-related genes via the abscisic acid (ABA) signaling pathway (Wang et al., 2018). The *ZmMYB-R1* gene is activated by cold, exogenous ABA, drought, heat, and high salinity (Campalans et al., 2001). Additionally, a mutant (*bzip60-2*) with reduced expression of *bZIP60* exhibited a diminished heat shock response at elevated temperatures and failed to normally upregulate a set of heat shock protein genes in response to increased temperature (Li et al., 2020a).

In recent years, high-throughput sequencing has emerged as a powerful tool for investigating the molecular mechanisms and biological traits of plants in response to abiotic stresses (Farooqi et al., 2022; Zhou et al., 2022; He et al., 2025; Liu et al., 2025). Numerous enriched pathways, including phenylpropanoid biosynthesis, flavonoid biosynthesis, and starch and sucrose metabolism, have been implicated in the heat stress response of maize, as revealed through transcriptome analysis (Wang et al., 2023; Liu et al., 2022a, Li et al., 2020b). Although RNA sequencing (RNA-seq) has identified numerous genes in maize under HT conditions (Farooqi et al., 2022), the understanding of transcriptomic changes during anthesis in maize under HT stress remains limited compared with other plants and tissues. Moreover, there is scant information regarding whether and how JA mediates the effects of HT stress on spikelet opening in maize inbred lines during anthesis. Consequently, this study aims to elucidate the role of JA in influencing seed setting and spikelet-opening rates in maize under HT stress through transcriptomic analysis. Our research enhances the understanding of molecular responses to HT stress in maize, contributing to the development of heat-tolerant maize varieties.

## Materials and methods

### Plant materials and treatments

For this study, we selected Chang 7-2 (a heat-tolerant maize inbred line) and Yu 727 and Y8201 (heat-sensitive maize inbred lines) due to their contrasting performances during tassel flowering under HT conditions, as observed in previous studies (Liu et al., 2022b). The duration to anthesis for the three maize lines was recorded as follows: C7 required 68 days, Y7 required 75 days, and 8201 required 78 days. Three inbred lines were designated as C7, Y7, and Y8 and were potted at the Chongqing Academy of Agricultural Sciences (29°49′43″ N, 106°21′13″ E), located in Baishiyi Town, Jiulongpo District, Chongqing City. Uniformity was ensured by selecting seedlings at the V3 stage. Each 20-m² plot contained 120 plants spaced at 80 × 20 cm, with adjacent plots separated by 1 m to minimize pollen-mediated effects on seed set. The experiment employed a randomized block design with three replicates. Plants were fully irrigated throughout the growth period. Spraying experiments were conducted to investigate the effects of MeJA (methyl jasmonic acid) on enhancing seed setting and spikelet opening under HT stress. Maize spikelets were treated with ultrapure water (control, CK) or MeJA (1 mM, JA). MeJA treatment was commenced subsequent to the complete emergence of the tassel from the uppermost leaves. A single application of 30 mL per plant was delivered using a sprayer between 07:00 and 12:00, with the application occurring once every hour. Heat stress was induced at a temperature of 38°C, whereas the normal growing temperature conditions prior to treatment were maintained at 32°C. For each of the six sample seedlings, 20 spikelets were combined to form a single biological sample, resulting in three sets of samples. Maize spikelets were collected on the day of anthesis and assigned sample names C7_CK, Y7_CK, Y7_JA, Y8_CK, and Y8_JA. Immediately after collection, the spikelets were frozen in liquid nitrogen, transported on dry ice, and stored at −80°C for subsequent analysis.

### RNA sequencing and qRT-PCR analysis

Total RNA was extracted from plant samples using the RNAprep Pure Plant Kit (DP441, Tiangen Technologies, Beijing, China) according to the manufacturer’s protocol. RNA quality and purity were evaluated using the Agilent 2100 Bioanalyzer (Agilent Technologies, CA, USA) and the NanoDrop ND-2000 Spectrophotometer (NanoDrop Technologies). Only RNA samples meeting stringent quality criteria (OD260/280 ratio: 1.8–2.2; OD260/230 ratio: ≥2.0; RIN: ≥6.5; 28S:18S ratio: ≥1.0; quantity: >1 μg) were selected for subsequent experiments. Illumina RNA-seq libraries were constructed from 1 μg of RNA per sample using the TruSeq™ RNA Sample Preparation Kit (Illumina), following the manufacturer’s recommended protocol. The resulting libraries were further evaluated using the Agilent 2100 Bioanalyzer (Agilent Technologies, CA, USA). Qualified libraries were subsequently sequenced on the Illumina NovaSeq 6000 platform at Norminkoda Technologies (Wuhan, China).

Raw paired-end reads were trimmed and quality-filtered using SeqPrep and Sickle with default parameters. The resulting high-quality reads were then mapped to the maize reference genome (Zm-B73-REFERENCE-NAM-5.0) using HISAT2 (Kim et al., 2015) and assembled with StringTie (Pertea et al., 2015). Gene expression values were quantified as reads per kilobase of transcript per million mapped reads (FPKM) for all genes. Differential expression analysis was performed using DESeq2, with DEGs identified based on thresholds of |log2FoldChange| > 1 and Q-value ≤ 0.05 (Love et al., 2014). Kyoto Encyclopedia of Genes and Genomes (KEGG) pathway enrichment analysis was applied using the web service (https://www.omicshare.com/tools/).

qRT-PCR was performed on a CFX96TM Real-Time System (Bio-Rad, USA) with 1× iQ™ SYBR Green Supermix (Bio-Rad, USA) according to the manual, and data were analyzed by the native software (Bio-Rad, USA). Actin gene *ZmACTIN* (Zm00001eb348450) served as the reference genes in maize. Gene-specific primers used for qRT-PCR are shown in **Supplementary Table 1**.

### Measurement of Pro and MDA content

The content of Pro was measured using the ninhydrin reaction method (Bates et al., 1973). Fresh leaf samples (0.1 g) were homogenized in 2.5 mL of 3% sulfosalicylic acid solution, followed by centrifugation at 10,000 rpm for 5 min. The supernatant (2 mL) was mixed with 2 mL acid ninhydrin and 2 mL glacial acetic acid and then incubated at 100 °C for 1 h. Subsequently, 4 mL toluene was added to extract the chromophore, and its absorbance was measured at 520 nm. Proline quantification was performed using a standard curve generated from proline solutions (10–100 ppm).

Fresh samples (~0.5 g) were ground in liquid nitrogen and homogenized with 4.5 mL of ice-cold phosphate-buffered saline (PBS, 0.1 M; pH 7.4). The homogenate was centrifuged at 4,000 × g for 10 min at 4 °C. MDA content was quantified using a commercial plant ELISA kit (colorimetric method, Nanjing Jiancheng Bioengineering Institute, Jiangsu, China) by measuring absorbance at 532 nm in a microplate reader.

### Determination of the activity of antioxidant enzymes

Catalase (CAT) and peroxidase (POD) activities were measured using established methods (Maehly and Chance, 1954). For CAT assay, a fixed amount of H_2_O_2_ was reacted with the enzyme sample, and the reaction was terminated after 1 min using a CAT-specific inhibitor. In the presence of peroxidase, residual H_2_O_2_ reacted with 3,5-dichloro-2-hydroxybenzenesulfonic acid and 4-aminophenazone to generate a chromophore, with absorbance intensity at 510 nm inversely proportional to the CAT concentration.

POD activity was assessed by preparing a reaction mixture containing 100 μL 0.5% H_2_O_2_, 100 μL 0.5% guaiacol, 1.8 mL phosphate buffer, and 100 μL enzyme extract. Absorbance at 470 nm was recorded every 30 s for 3 min, with one unit of POD activity defined as a 0.01 absorbance change per minute.

### Kernel number per ear and seed set

At physiological maturity, 10 central-section ears per plot were harvested. Kernel number per ear (KN/ear) was calculated by multiplying the row number per ear by the kernel number per row. For ears with low seed set, KN/ear was obtained through direct counting of all kernels after threshing. Seed set percentage was derived by dividing KN/ear by the total number of spikelets per ear.

### Statistics

Principal component analysis (PCA) and heat map were conducted using the web service (https://www.omicshare.com/tools/). HCA was performed using cloud tools of Norminkoda (http://nmkdcloud.com/). The column diagram was generated with GraphPad Prism 5. The analysis results for bidirectional grouping bar chart plots were generated using the CNSknowall platform (https://cnsknowall.com).

## Results

### The effect of JA on seed set under HT stress

**Figure 1:**
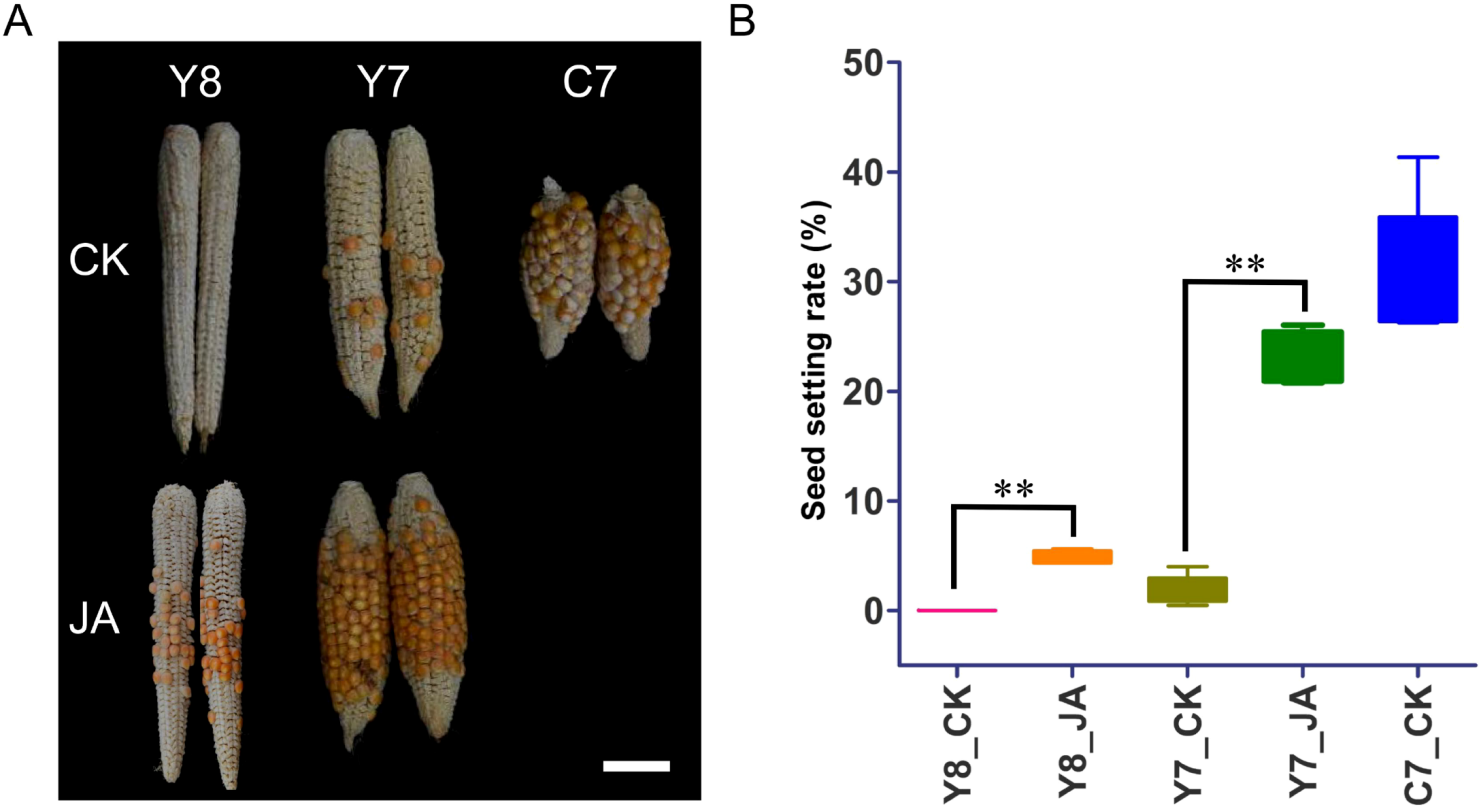
Effect of JA on seed set under HT stress. The inbred lines Y7, Y8, and C7 were subjected to either MeJA (1 mM) treatment (JA) or control (CK) conditions. **(A)** Representative images of mature ears at harvest. Bar = 5 cm. **(B)** Percentage seed set. Data are means (95% confidence interval) of 10 replicate plants. Significant differences between the control and HT treatments were determined using Student’s *t*-test: **P < 0.01.

To assess the effect of JA on seed set under HT stress, three maize inbred lines with varying levels of heat resistance were subjected to HT treatment in an experimental field. Maize spikelets were treated with MeJA around the time of anthesis. The heat tolerance coefficient was determined based on the seed setting rate (**Figure 1**). Under HT stress, kernel development in the two heat-sensitive varieties, Y7 and Y8, was significantly more inhibited compared with the heat-tolerant variety, C7. Furthermore, JA application resulted in higher seed setting rates in Y7 and Y8 compared with the control group under heat stress conditions. These findings suggest that JA spray application can enhance seed setting rates in maize under heat stress.

### JA enhances spikelet opening and spikelet opening angle under HT stress

**Figure 2:**
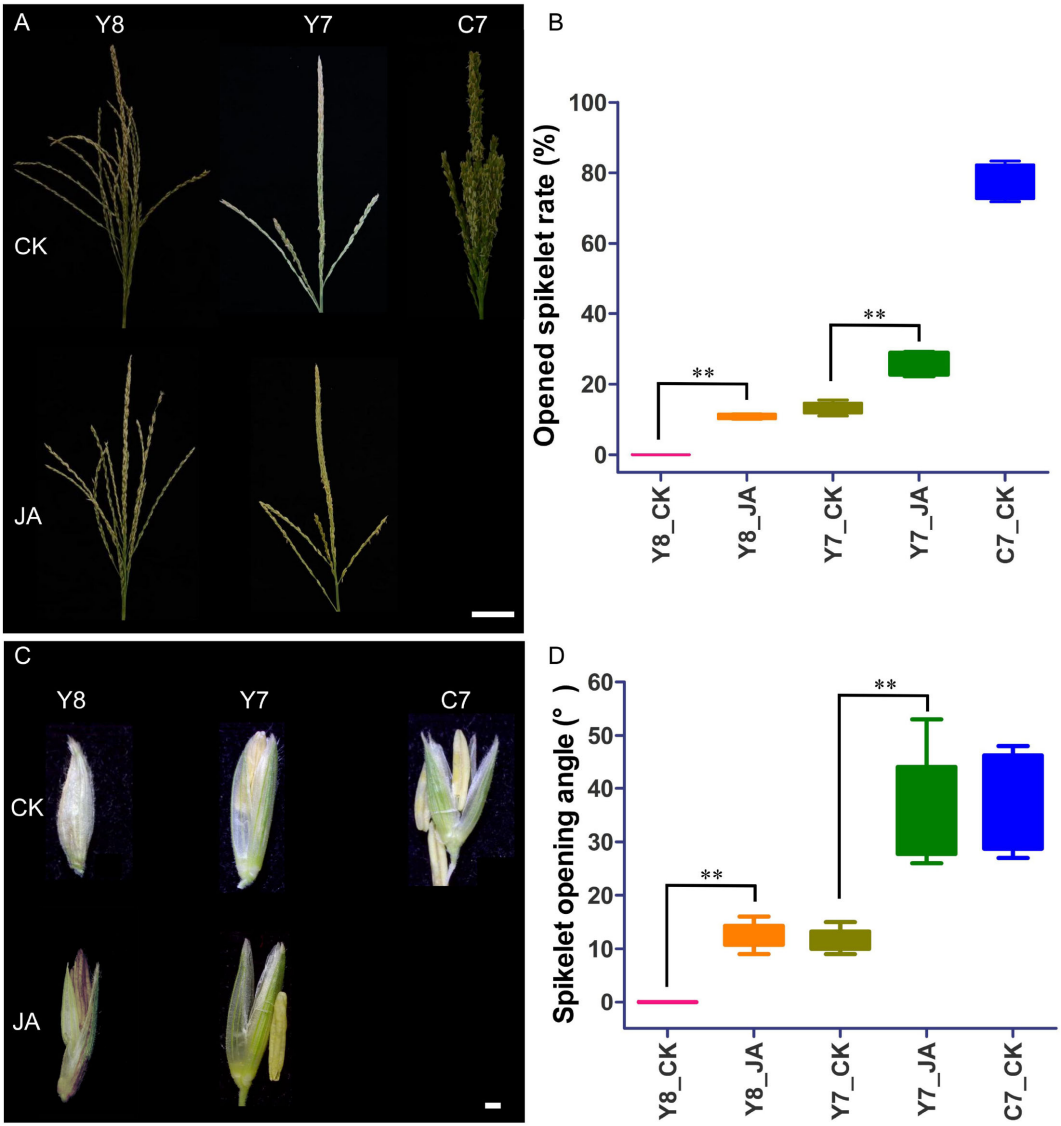
JA counteracts heat stress-induced inhibition of spikelet opening and spikelet opening angle in maize tassels. **(A)** Representative images of tassel images. CR, central rachis; TB, tassel branch. Bar = 5 cm. **(B)** Opened spikelet rate, determined as the mean value over the second and third days after anthesis. **(C)** Representative images of spikelets. Bar = 1 mm. **(D)** Spikelet opening angle. Data are means (95% confidence interval) of 10 replicate plants. Significant differences between the control and HT treatments were determined using Student’s *t*-test: **P < 0.01.

To evaluate the effect of JA on spikelet opening under heat stress, the rate of spikelet opening was calculated on the second day post-anthesis. Spikelet opening in the tassels of Y7 and Y8 was significantly impeded by HT stress, whereas C7 exhibited a markedly higher spikelet opening rate in response to HT compared with the other two cultivars. Additionally, JA application led to higher spikelet opening rates in Y7 and Y8 than in the control group under HT stress conditions. The results indicated that the application of JA via spraying could enhance the rate of opened spikelets in maize under HT conditions, as demonstrated in **Figures 2A, B**. On the third day post-anthesis, representative spikelets exhibiting the largest opening angles were collected from the central spikes of the tassels in each treatment group. Compared with the control group, the spikelet opening angles were increased in the Y7 and Y8 treatments following JA application (**Figures 2C, D**). This finding suggests that JA enhances spikelet opening angles under HT stress conditions.

### Promoted HT stress response physiological traits in maize treated with JA

**Figure 3:**
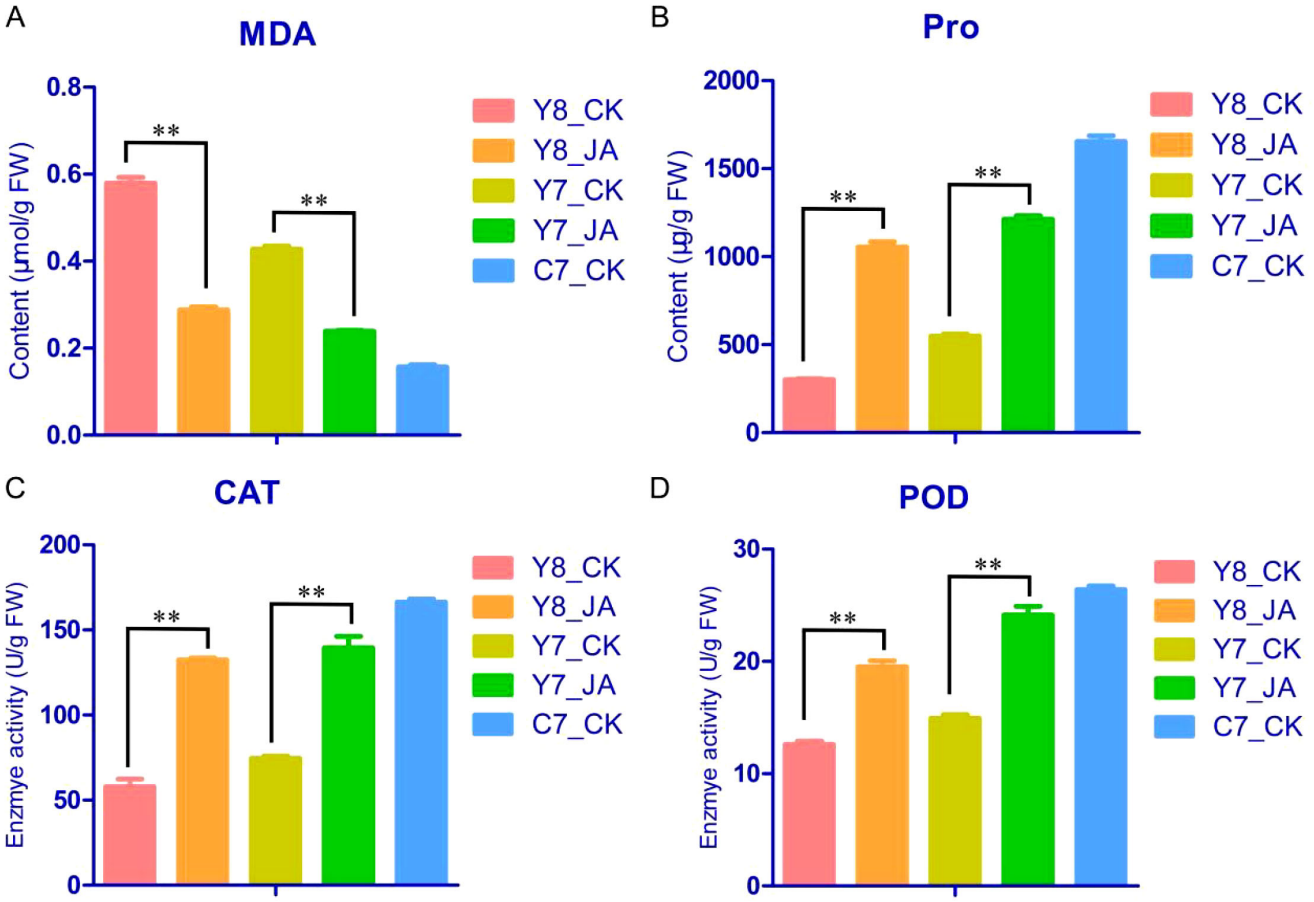
The content of MDA **(A)** and Pro **(B)**, and the activities of CAT **(C)** and POD **(D)** in the spikelet of maize treated with MeJA (JA) or water (CK) under HT stress. Data are presented as means ± SE (n = 3). The results were analyzed by Student’s *t*-test in a two-tailed analysis. Significance was defined as *p* < 0.01 (**).

In plant research, malondialdehyde (MDA), proline (Pro), catalase (CAT), and peroxidase (POD) are crucial physiological indices commonly used to assess plant stress responses. Consequently, these four physiological indicators were measured in this study (**Figure 3**). Under HT stress, the MDA content in the C7 treatment was lower than that in Y7 and Y8. Following JA treatment, MDA levels in both Y7 and Y8 significantly decreased compared with the control (**Figure 3A**). Furthermore, C7 exhibited significantly higher Pro contents compared with Y7 and Y8 (**Figure 3B**), along with elevated CAT and POD enzyme activities (**Figures 3C, D**). Notably, JA treatment led to significantly increased levels of Pro, CAT, and POD in Y7 and Y8 compared with the control (**Figure 3**). These results suggested that exogenous JA activated the antioxidant systems and improved the antioxidant capacities of spikelet under HT stress.

### Transcriptome analysis reveals the pathway regulated by JA under HT stress

**Figure 4:**
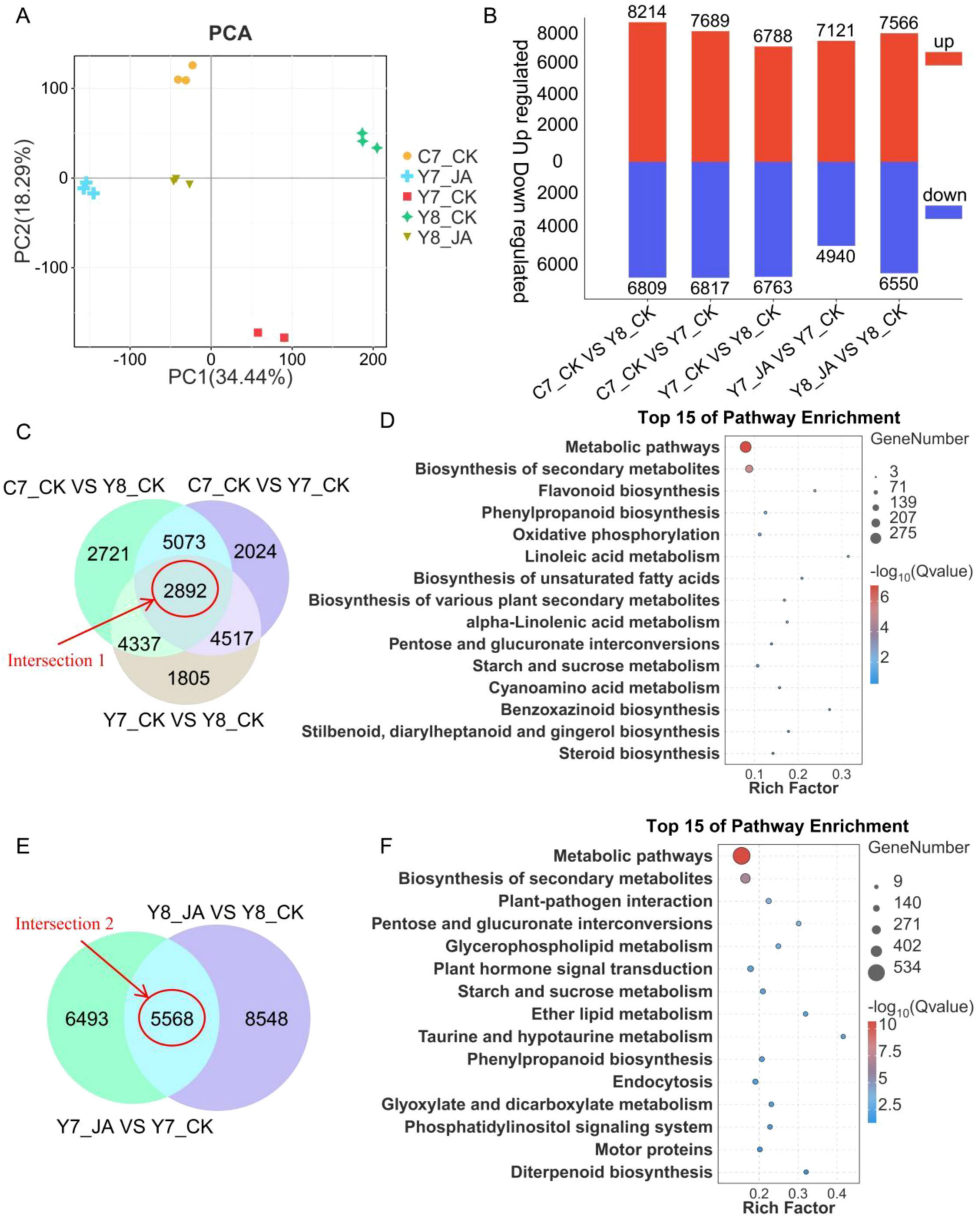
An overview of transcriptome and DEG analyses. **(A)** PCA score plots of genes identified based on FPKM values. **(B)** Overview of DEGs between comparison groups. **(C, E)** Venn diagrams depicting DEG counts across comparison groups, with each red line indicating the intersections among the comparison groups. The control group displayed on the right side of each comparison. **(D, F)** The rich factor plot of the KEGG pathway enrichment analysis results. The ordinate represents the name of the pathway, the size of the dot represents the number of genes, and the color represents the Q value.

Using Illumina paired-end RNA-seq, 15 samples yielded 0.9 billion high-quality reads after filtering low-quality data (**Supplementary Table 1**). Each library produced >44 million clean reads, with 95% Q30 bases and ~55% GC content. Mapping rates to the maize reference genome (Zm-B73-REFERENCE-NAM-5.0, GCF_902167145.1) ranged from 70.03% to 84.05% (**Supplementary Table 1**). FPKM-normalized expression analysis identified 46,176 expressed genes across all samples (**Supplementary Table 2**). PCA was applied to all transcriptome data to determine the associated variation. PCA of transcriptome data revealed that PC1 (34.44%) and PC2 (18.29%) accounted for 52.73% of total variation (**Figure 4A**). Biological replicates clustered tightly, corroborated by hierarchical clustering analysis (HCA) (**Supplementary Figure 1**), confirming data reproducibility (**Figure 2A**).

To determine which genes were related to the HT stress response and JA induction, DEGs between the five treatments were identified. A total of 28,155 DEGs in the five comparisons (Y7_CK vs. Y8_CK, Y7_CK vs. C7_CK, Y8_CK vs. C7_CK, Y7_CK vs. Y7_JA, and Y8_CK vs. Y8_JA) were identified (**Figure 4B**; **Supplementary Table 3**). Venn analysis was conducted to further characterize DEGs. The comparisons of Y7_CK vs. Y8_CK, Y7_CK vs. C7_CK, and Y8_CK vs. C7_CK were examined, revealing 2,892 common genes (intersection 1) that were differentially expressed across these comparisons (**Figure 4C**), whereas Venn analysis revealed 5,568 common DEGs (intersection 2) between the comparisons of Y7_CK vs. Y7_JA and Y8_CK vs. Y8_JA (**Figure 4E**). Further KEGG enrichment analyses were conducted to examine the role of DEGs (**Supplementary Table 4**). The most significantly enriched pathways are primarily associated with metabolism, including phenylpropanoid biosynthesis, flavonoid biosynthesis, starch and sucrose metabolism, and other pathways (**Figures 4D, F**).

### Identification of the candidate TFs regulated by JA under TH stress

**Figure 5:**
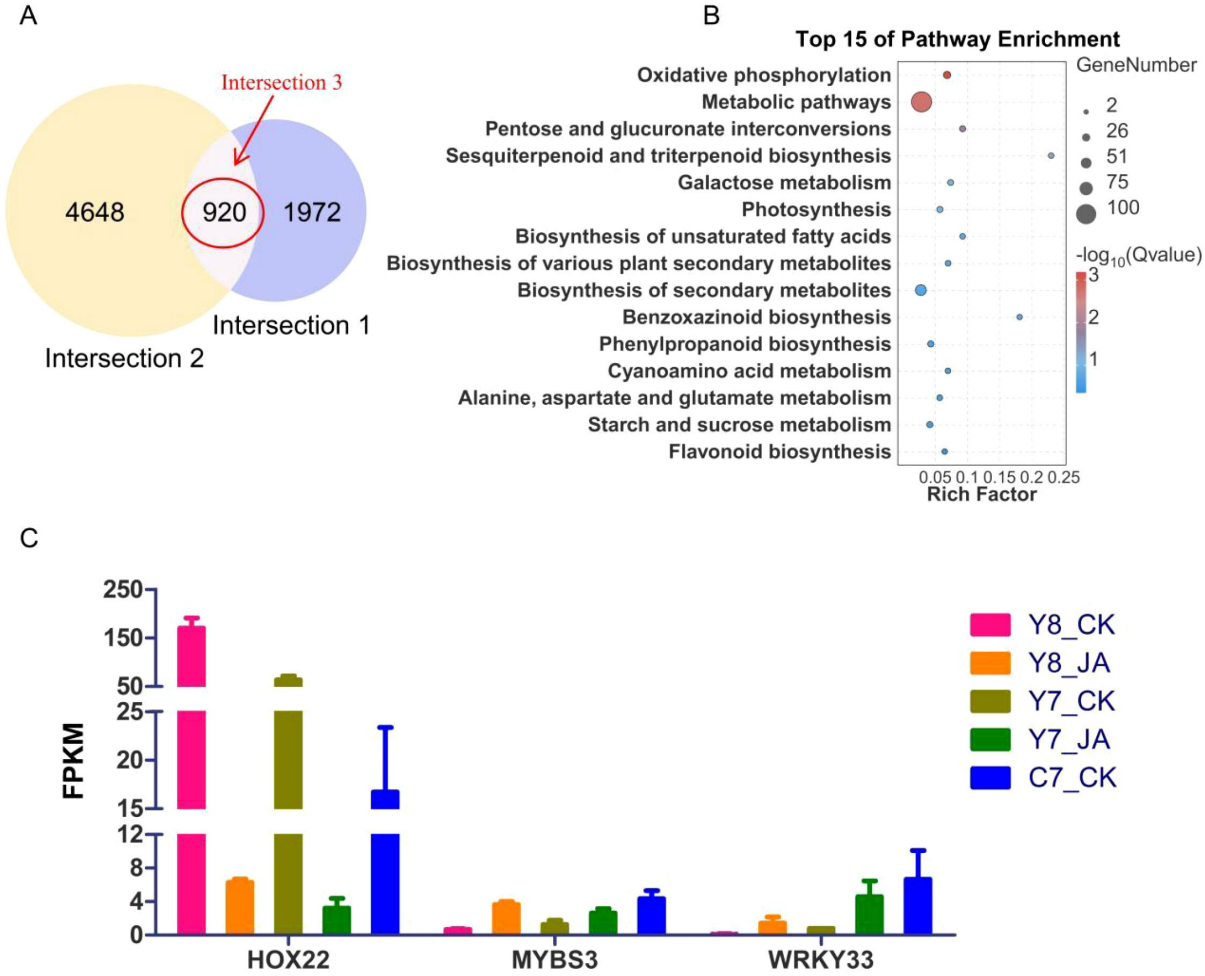
**(A)** Venn diagrams depicting DEG counts across comparison groups, with each red line indicating the intersections among the two comparison groups. The control group displayed on the right side of each comparison. **(B)** Rich factor plot of the KEGG pathway enrichment analysis results. The ordinate represents the name of the pathway, the size of the dot represents the number of genes, and the color represents the Q value. **(C)** Expression levels of three candidate TF genes.

To refine the candidate gene pool, comparative analysis of intersection 1 and intersection 2 datasets identified 920 common DEGs (intersection 3) (**Figure 5A**). KEGG enrichment analysis revealed that the most significantly enriched pathways were phenylpropanoid biosynthesis, flavonoid biosynthesis, and starch and sucrose metabolism, among others (**Figure 5B**).

From the 920 common DEGs, 42 TFs were identified (**Supplementary Table 5**). Expression profiling of these TFs (**Supplementary Figure 2**) highlighted HOX22 (negative regulation), MYBS3, and WRKY33 (positive regulation) as potential candidates associated with HT stress response (**Figure 5C**). The trend in the expression levels of *HOX22*, *MYBS3*, and *WRKY33* from transcriptome data aligned with the qRT-PCR results (**Supplementary Figure 3**).

## Discussion

### JA mitigates heat-induced yield losses

Global food security is increasingly threatened by the rising frequency and intensity of heatwaves (Zhao et al., 2017; Magaña Ugarte et al., 2019; Tigchelaar et al., 2018). Maize yields are particularly vulnerable to climate-induced warming, with projected declines attributed to prolonged heat exposure in major agricultural regions (Li and Howell, 2021; Wang et al., 2020b). JA, a key phytohormone, has demonstrated critical roles in plant stress responses and developmental processes, including floret formation and spikelet maturation (Turner et al., 2002; Trang Nguyen et al., 2019; Wani et al., 2016). Notably, JA signaling enhances heat tolerance in Arabidopsis (Clarke et al., 2009) and regulates rice spikelet development (Kim et al., 2009). This study investigates the effects of JA on maize seed setting and spikelet opening under heat stress.

HT stress severely reduces maize seed set, compromising grain yield (Borrás and Vitantonio-Mazzini, 2018; Deryng et al., 2014). Our findings align with prior research: heat-sensitive varieties (Y7, Y8) showed greater kernel development inhibition than the tolerant variety (C7) (**Figure 1**). Exogenous JA application improved seed set rates in both Y7 and Y8 under HT stress in maize (**Figure 1**), mirroring its beneficial effects in rice (Pak et al., 2021). These results indicated that spray application of JA could enhance the seed setting rate in maize under heat stress conditions.

### JA mitigates heat-induced spikelet dysfunction in maize

Contrasting effects of temperature on spikelet behavior were observed: Moderate heat accelerates opening in rice, whereas extreme heatwaves induce closure (Cai et al., 2014). This phenomenon poses a greater threat to maize seed set due to the spatial separation between tassels and ears, making pollen transfer highly sensitive to spikelet opening impairment (Chen et al., 2020). In this study, HT stress significantly inhibited the spikelet opening rate and angle in tassels of heat-sensitive cultivars Y7 and Y8, whereas heat-tolerant cultivar C7 exhibited a markedly higher opening rate and angle under HT conditions. Moreover, exogenous JA application increased the spikelet opening rates and angle in Y7 and Y8 compared with the controls under HT stress (**Figures 2A, B**). Our findings demonstrate that exogenous JA application significantly enhances both spikelet opening rate and angle in HT-stressed maize (**Figures 2A, B**), confirming JA’s protective role against HT stress.

### JA enhances antioxidant defense mechanisms under HT stress

To mitigate the detrimental effects of HT environments, plants have evolved a range of adaptive mechanisms to endure adverse conditions. Under HT stress, reactive oxygen species (ROS) generated within plant tissues induce varying levels of oxidative stress (Ruan et al., 2024). Consequently, the activities of CAT and POD enzymes, along with the concentrations of MDA and Pro, increase correspondingly (Mittler et al., 2012; Guo et al., 2024). Specifically, under HT stress, the MDA content in genotype C7 was observed to be lower than that in genotypes Y7 and Y8. Following treatment with JA, MDA levels in both Y7 and Y8 were significantly reduced compared with the control group (**Figure 3A**). Furthermore, the C7 genotype demonstrated higher CAT and POD enzyme activities, as well as elevated Pro content, relative to Y7 and Y8. Post-JA treatment, both Y7 and Y8 exhibited significant enhancements in CAT and POD activities and Pro content compared with their untreated counterparts (**Figure 3**). These findings suggest that genotype C7 possesses superior antioxidant capacity compared with Y7 and Y8 and that JA treatment augments antioxidant activity in both Y7 and Y8.

### Transcriptomic insights into JA-mediated thermotolerance

In recent years, high-throughput sequencing has emerged as a powerful tool for elucidating the molecular mechanisms and biological traits of plants in response to abiotic stresses (Farooqi et al., 2022; Zhou et al., 2022). Numerous genes in maize have been identified using RNA-seq under HT conditions (Farooqi et al., 2022). To elucidate the molecular basis of heat stress resistance, transcriptome profiling was performed to investigate the jasmonic acid (JA)-mediated regulatory pathways associated with thermotolerance. Transcriptomic analysis indicated that under HT stress, three KEGG pathways—phenylpropanoid biosynthesis, flavonoid biosynthesis, and starch and sucrose metabolism—potentially contribute to JA-mediated heat stress resistance (**Figures 4D, F**, **5B**; **Supplementary Table 4**). These findings are consistent with previous studies (Wang et al., 2023; Liu et al., 2022a, Li et al., 2020b).

Furthermore, earlier research has emphasized the significant role of transcriptional regulatory mechanisms in mediating adaptive responses to heat stress in plants. Heat stress triggers the TF-mediated expression of various genes in plants, including those encoding HSF, bZIP, NAC, MYB, and WRKY proteins (Cheng et al., 2021; Scharf et al., 2012; Ruan et al., 2024; El-Sappah et al., 2022). Our analysis identified 42 transcription factors that were differentially expressed, suggesting their potential regulatory roles in the observed responses (**Supplementary Figure 2**, **Supplementary Table 5**). Through the analysis of the expression characteristics of these 42 TFs, we identified one negative regulatory factor (HOX22) and two positive regulators (MYBS3 and WRKY33) involved in JA-mediated HT stress resistance in maize. Further studies are now needed to investigate the relationships between those genes and heat stress response in maize. Our research will be facilitated to identify essential heat tolerance genes in maize, thereby contributing to breeding heat resistance maize varieties.

## Data Availability

The data presented in the study are deposited in the NCBI (National Center for Biotechnology Information) repository, accession number PRJNA1422265.

## References

[B1] Acosta I. F. Przybyl M. (2019). Jasmonate signaling during Arabidopsis stamen maturation. Plant Cell Physiol. 60, 2648–2659. doi: 10.1093/pcp/pcz201, PMID: 31651948 PMC6896695

[B2] Bates L. S. Waldren R. P. Teare I. D. (1973). Rapid determination of free proline for water-stress studies. Plant Soil 39, 205–207. doi: 10.1007/BF00018060, PMID: 41758449

[B3] Borrás L. Vitantonio-Mazzini L. N. (2018). Maize reproductive development and kernel set under limited plant growth environments. J. Exp. Bot. 69, 3235–3243. doi: 10.1093/jxb/erx452, PMID: 29304259

[B4] Cai Q. Yuan Z. Chen M. Yin C. Luo Z. Zhao X. . (2014). Jasmonic acid regulates spikelet development in rice. Nat. Commun. 5, 3476. doi: 10.1038/ncomms4476, PMID: 24647160

[B5] Campalans A. Pagès M. Messeguer R. (2001). Identification of differentially expressed genes by the cDNA-AFLP technique during dehydration of almond (Prunus amygdalus). Tree Physiol. 21, 633–643. doi: 10.1093/treephys/21.10.633, PMID: 11446992

[B6] Chen J. Xu Y. Fei K. Wang R. He J. Fu L. . (2020). Physiological mechanism underlying the effect of high temperature during anthesis on spikelet-opening of photo-thermo-sensitive genic male sterile rice lines. Sci. Rep. 10, 2210. doi: 10.1038/s41598-020-59183-0, PMID: 32042005 PMC7010791

[B7] Cheng Z. Luan Y. Meng J. Sun J. Tao J. Zhao D. (2021). WRKY transcription factor response to high-temperature stress. Plants (Basel) 10, 2211. doi: 10.3390/plants10102211, PMID: 34686020 PMC8541500

[B8] Clarke S. M. Cristescu S. M. Miersch O. Harren F. J. M. Wasternack C. Mur L. A. J. (2009). Jasmonates act with salicylic acid to confer basal thermotolerance in Arabidopsis thaliana. New Phytol. 182, 175–187. doi: 10.1111/j.1469-8137.2008.02735.x, PMID: 19140948

[B9] Dafni A. Hesse M. Pacini E. (2000). From anther and pollen ripening to pollen presentation (Vienna: Springer Vienna), 19–43. doi: 10.1007/978-3-7091-6306-1, PMID:

[B10] Deryng D. Conway D. Ramankutty N. Price J. Warren R. (2014). Global crop yield response to extreme heat stress under multiple climate change futures. Environ. Res. Lett. 9, 34011. doi: 10.1088/1748-9326/9/3/034011

[B11] Dresselhaus T. Franklin-Tong N. (2013). Male-female crosstalk during pollen germination, tube growth and guidance, and double fertilization. Mol. Plant 6, 1018–1036. doi: 10.1093/mp/sst061, PMID: 23571489

[B12] El-Sappah A. H. Rather S. A. Wani S. H. Elrys A. S. Bilal M. Huang Q. . (2022). Heat stress-mediated constraints in maize (Zea mays) production: challenges and solutions. Front. Plant Sci. 13. doi: 10.3389/fpls.2022.879366, PMID: 35615131 PMC9125997

[B13] Farooqi M. Q. U. Nawaz G. Wani S. H. Choudhary J. R. Rana M. Sah R. P. . (2022). Recent developments in multi-omics and breeding strategies for abiotic stress tolerance in maize (Zea mays L.). Front. Plant Sci. 13. doi: 10.3389/fpls.2022.965878, PMID: 36212378 PMC9538355

[B14] Guo J. Wang Z. Li J. Qu L. Chen Y. Li G. . (2024). Salicylic acid promotes endosperm development and heat-tolerance of waxy maize (Zea mays L. var. ceratina Kulesh) under heat stress. Plant Stress 14, 100684. doi: 10.1016/j.stress.2024.100684, PMID: 41756733

[B15] Haider S. Iqbal J. Naseer S. Yaseen T. Shaukat M. Bibi H. . (2021). Molecular mechanisms of plant tolerance to heat stress: current landscape and future perspectives. Plant Cell Rep. 40, 2247–2271. doi: 10.1007/s00299-021-02696-3, PMID: 33890138

[B16] He Y. Deng Z. He S. Qi Z. Chang H. Liu P. . (2025). Transcriptome and co-expression network analysis reveal the genetic basis of cell wall components in maize stalks. BMC Genomics 26, 620. doi: 10.1186/s12864-025-11816-2, PMID: 40597629 PMC12210581

[B17] Hou F. Liang Y. Sang M. Zhao G. Song J. Liu P. . (2025). Complex regulatory network of ZmbZIP54-mediated Pb tolerance in maize. Plant Physiol. Biochem. 224, 109945. doi: 10.1016/j.plaphy.2025.109945, PMID: 40279841

[B18] Keijzer C. J. Reinders M. C. Leferink-Ten K. H. B. (1996). The mechanics of the grass flower: the extension of the staminal filaments and the lodicules of maize. Ann. Bot., 675–683. doi: 10.1006/anbo.1996.0084, PMID: 39885891

[B19] Kim E. H. Kim Y. S. Park S. H. Koo Y. J. Choi Y. D. Chung Y. Y. . (2009). Methyl jasmonate reduces grain yield by mediating stress signals to alter spikelet development in rice. Plant Physiol 149, 1751–1760. doi: 10.1104/pp.108.34684 19211695 PMC2663756

[B20] Kim D. Langmead B. Salzberg S. L. (2015). HISAT: a fast spliced aligner with low memory requirements. Nat. Methods 12, 357–360. doi: 10.1038/nmeth.3317, PMID: 25751142 PMC4655817

[B21] Li Z. Howell S. H. (2021). Heat stress responses and thermotolerance in maize. Int. J. Mol. Sci. 22, 948. doi: 10.3390/ijms22020948, PMID: 33477941 PMC7833377

[B22] Li Z. Tang J. Srivastava R. Bassham D. C. Howell S. H. (2020a). The transcription factor bZIP60 links the unfolded protein response to the heat stress response in maize. Plant Cell 32, 3559–3575. doi: 10.1105/tpc.20.00260, PMID: 32843434 PMC7610289

[B23] Li Y. Wang X. Li Y. Zhang Y. Gou Z. Qi X. . (2020b). Transcriptomic analysis revealed the common and divergent responses of maize seedling leaves to cold and heat stresses. Genes 11, 881. doi: 10.3390/genes11080881, PMID: 32756433 PMC7464670

[B24] Liu M. Sheng D. Liu X. Wang Y. Hou X. Wang Y. . (2022b). Dissecting heat tolerance and yield stability in maize from greenhouse and field experiments. J. Agron. Crop Sci./Zeitschrift fur acker-und pflanzenbau 208, 348–361. doi: 10.1111/jac.12590, PMID: 41744481

[B25] Liu M. Yang Y. Liang T. Hou F. Zhang M. He S. . (2025). Dynamic transcriptome and GWAS uncover a hydroxyproline-rich glycoprotein that suppresses Agrobacterium-mediated transformation in maize. Mol. Plant 18, 747–764. doi: 10.1016/j.molp.2025.03.011, PMID: 40114443

[B26] Liu J. Zhang L. Huang L. Yang T. Ma J. Yu T. . (2022a). Uncovering the gene regulatory network of maize hybrid ZD309 under heat stress by transcriptomic and metabolomic analysis. Plants 11, 677. doi: 10.3390/plants11050677, PMID: 35270147 PMC8912342

[B27] Love M. I. Huber W. Anders S. (2014). Moderated estimation of fold change and dispersion for RNA-seq data with DESeq2. Genome Biol. 15, 550. doi: 10.1186/s13059-014-0550-8, PMID: 25516281 PMC4302049

[B28] Maehly A. C. Chance B. (1954). The assay of catalases and peroxidases. Methods Biochem. Anal. 1, 357–424. doi: 10.1002/9780470110171.ch14, PMID: 13193536

[B29] Magaña Ugarte R. Escudero A. Gavilán R. G. (2019). Metabolic and physiological responses of Mediterranean high-mountain and alpine plants to combined abiotic stresses. Physiol. Plant 165, 403–412. doi: 10.1111/ppl.12898, PMID: 30536685

[B30] Mittler R. Finka A. Goloubinoff P. (2012). How do plants feel the heat? Trends Biochem. Sci. 37, 118–125. doi: 10.1016/j.tibs.2011.11.007, PMID: 22236506

[B31] Pak H. Wang H. Kim Y. Song U. Tu M. Wu D. . (2021). Creation of male-sterile lines that can be restored to fertility by exogenous methyl jasmonate for the establishment of a two-line system for the hybrid production of rice (Oryza sativa L.). Plant Biotechnol. J. 19, 365–374. doi: 10.1111/pbi.13471, PMID: 32860735 PMC7868980

[B32] Pertea M. Pertea G. M. Antonescu C. M. Chang T. C. Mendell J. T. Salzberg S. L. (2015). StringTie enables improved reconstruction of a transcriptome from RNA-seq reads. Nat. Biotechnol. 33, 290–295. doi: 10.1038/nbt.3122, PMID: 25690850 PMC4643835

[B33] Prasad P. V. V. Boote K. J. Allen L. H. (2006). Adverse high temperature effects on pollen viability, seed-set, seed yield and harvest index of grain-sorghum [Sorghum bicolor (L.) Moench] are more severe at elevated carbon dioxide due to higher tissue temperatures. Agric. For. Meteorol. 139, 237–251. doi: 10.1016/j.agrformet.2006.07.003, PMID: 41756733

[B34] Prasad P. V. V. Djanaguiraman M. (2014). Response of floret fertility and individual grain weight of wheat to high temperature stress: sensitive stages and thresholds for temperature and duration. Funct. Plant Biol. 41, 1261–1269. doi: 10.1071/fp14061, PMID: 32481075

[B35] Ruan M. Zhao H. Wen Y. Chen H. He F. Hou X. . (2024). The complex transcriptional regulation of heat stress response in maize. Stress Biol. 4, 24. doi: 10.1007/s44154-024-00165-x, PMID: 38668992 PMC11052759

[B36] Saini N. Nikalje G. C. Zargar S. M. Suprasanna P. (2022). Molecular insights into sensing, regulation and improving of heat tolerance in plants. Plant Cell Rep. 41, 799–813. doi: 10.1007/s00299-021-02793-3, PMID: 34676458

[B37] Sánchez B. Rasmussen A. Porter R. (2014). Temperatures and the growth and development of maize and rice: a review. Glob. Chang. Biol. 20, 408–417. doi: 10.1111/gcb.12389, PMID: 24038930

[B38] Scharf K. D. Berberich T. Ebersberger I. Nover L. (2012). The plant heat stress transcription factor (Hsf) family: structure, function and evolution. Biochim. Biophys. Acta 1819, 104–119. doi: 10.1016/j.bbagrm.2011.10.002, PMID: 22033015

[B39] Shi W. Ishimaru T. Gannaban R. B. Oane W. Jagadish S. V. K. (2015). Popular Rice (Oryza sativa L.) Cultivars Show Contrasting Responses to Heat Stress at Gametogenesis and Anthesis. Crop Sci. 55, 589–596. doi: 10.2135/cropsci2014.01.0054

[B40] Teng L. Qing L. Shumei W. Xuepeng Z. Yuanquan C. Wangsheng G. . (2025). High temperature effects on maize photosynthesis during stress and recovery phase at the seed setting stage. BMC Plant Biol. 25, 454. doi: 10.1186/s12870-025-06047-2, PMID: 40211142 PMC11983829

[B41] Tigchelaar M. Battisti D. S. Naylor R. L. Ray D. K. (2018). Future warming increases probability of globally synchronized maize production shocks. Proc. Natl. Acad. Sci. U.S.A. 115, 6644–6649. doi: 10.1073/pnas.1718031115, PMID: 29891651 PMC6042138

[B42] Trang Nguyen H. Thi Mai To H. Lebrun M. Bellafiore S. Champion A. (2019). Jasmonates-the master regulator of rice development, adaptation and defense. Plants (Basel) 8, 339. doi: 10.3390/plants8090339, PMID: 31505882 PMC6784130

[B43] Turner J. G. Ellis C. Devoto A. (2002). The jasmonate signal pathway. Plant Cell 14 Suppl, S153–S164. doi: 10.1105/tpc.000679, PMID: 12045275 PMC151253

[B44] Vara Prasad P. V. Craufurd P. Q. Kakani V. G. (2001). Influence of high temperature during pre- and post-anthesis stages of floral development on fruit-set and pollen germination in peanut. Funct. Plant Biol. 28, 233–240. doi: 10.1071/pp00127, PMID: 41161682

[B45] Wang C. T. Ru J. N. Liu Y. W. Li M. Zhao D. Yang J. F. . (2018). Maize WRKY transcription factor zmWRKY106 confers drought and heat tolerance in transgenic plants. Int. J. Mol. Sci. 19, 3046. doi: 10.3390/ijms19103046, PMID: 30301220 PMC6213049

[B46] Wang Y. Sheng D. Zhang P. Dong X. Huang S. (2020a). High temperature sensitivity of kernel formation in different short periods around silking in maize. Environ. Exp. Bot. 183, 104343. doi: 10.1016/j.envexpbot.2020.104343, PMID: 41756733

[B47] Wang Y. Tao H. Zhang P. Hou X. Sheng D. Tian B. . (2020b). Reduction in seed set upon exposure to high night temperature during flowering in maize. Physiol. Plant 169, 73–82. doi: 10.1111/ppl.13049, PMID: 31747055

[B48] Wang Z. Xiao Y. Chang H. Sun S. Wang J. Liang Q. . (2023). The Regulatory Network of Sweet Corn (Zea mays L.) Seedlings under Heat Stress Revealed by Transcriptome and Metabolome Analysis. Int. J. Mol. Sci. 24, 10845. doi: 10.3390/ijms241310845, PMID: 37446023 PMC10341527

[B49] Wani S. H. Kumar V. Shriram V. Sah S. K. (2016). Phytohormones and their metabolic engineering for abiotic stress tolerance in crop plants. Crop J 4, 162–176. doi: 10.1016/j.cj.2016.01.010, PMID: 41756733

[B50] Yang Z. Cao Y. Shi Y. Qin F. Jiang C. Yang S. (2023). Genetic and molecular exploration of maize environmental stress resilience: Toward sustainable agriculture. Mol. Plant 16, 1496–1517. doi: 10.1016/j.molp.2023.07.005, PMID: 37464740

[B51] Yuan Z. Zhang D. (2015). Roles of jasmonate signalling in plant inflorescence and flower development. Curr. Opin. Plant Biol. 27, 44–51. doi: 10.1016/j.pbi.2015.05.024, PMID: 26125498

[B52] Zhang B. Wu S. Zhang Y. Xu T. Guo F. Tang H. . (2016). A High Temperature-Dependent Mitochondrial Lipase EXTRA GLUME1 Promotes Floral Phenotypic Robustness against Temperature Fluctuation in Rice (Oryza sativa L.). PloS Genet. 12, e1006152. doi: 10.1371/journal.pgen.1006152, PMID: 27367609 PMC4930220

[B53] Zhao C. Liu B. Piao S. Wang X. Lobell D. B. Huang Y. . (2017). Temperature increase reduces global yields of major crops in four independent estimates. Proc. Natl. Acad. Sci. U.S.A. 114, 9326–9331. doi: 10.1073/pnas.1701762114, PMID: 28811375 PMC5584412

[B54] Zhou R. Jiang F. Niu L. Song X. Yu L. Yang Y. . (2022). Increase crop resilience to heat stress using omic strategies. Front. Plant Sci. 13. doi: 10.3389/fpls.2022.891861, PMID: 35656008 PMC9152541

