# Supplementary material for: Transcriptome analysis reveals the role of jasmonate in regulating maize spikelet opening and seed set under high temperature stress
Source: Front Plant Sci. 2026 Mar 4;17:1710459. doi: 10.3389/fpls.2026.1710459 (PMC12995804; doi:10.3389/fpls.2026.1710459)
Supplement: Supplementary file 1 [file Supplementaryfile1.zip › Supplementary materals.docx]

**Supplementary Materials**


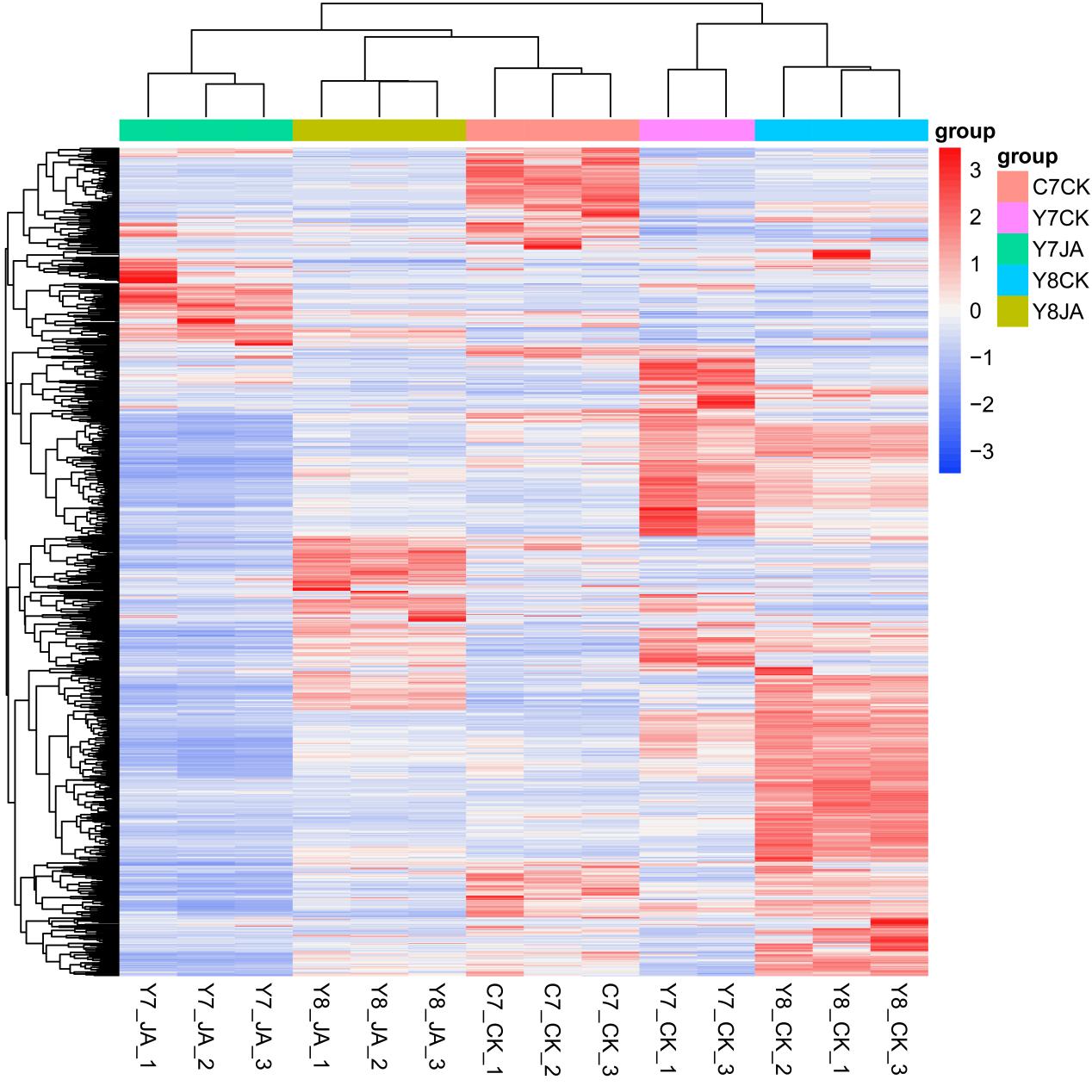


**Figure S1.** HCA analysis.


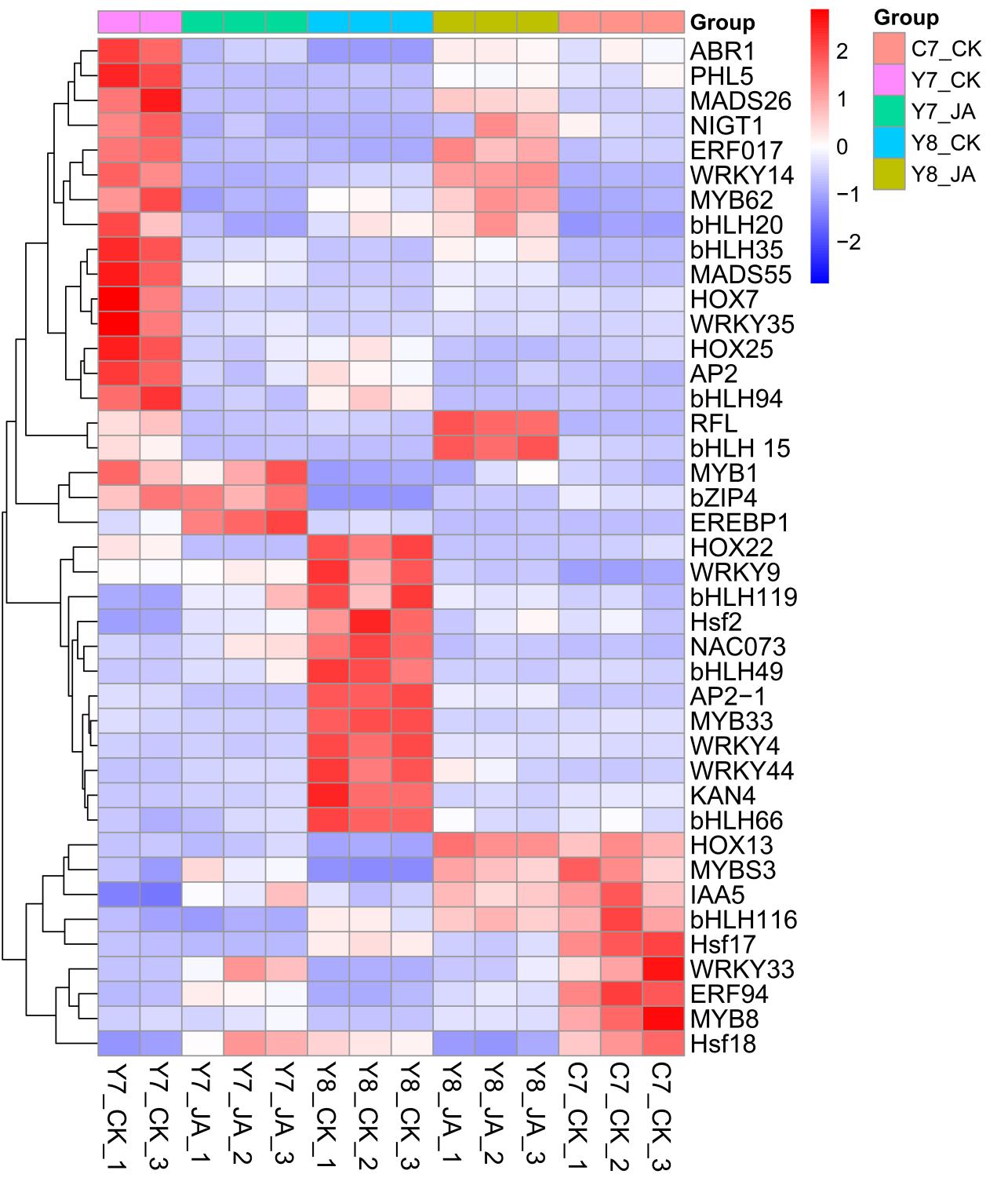


**Figure S2.** Heatmap of 42 TFs originated from intersection 3.


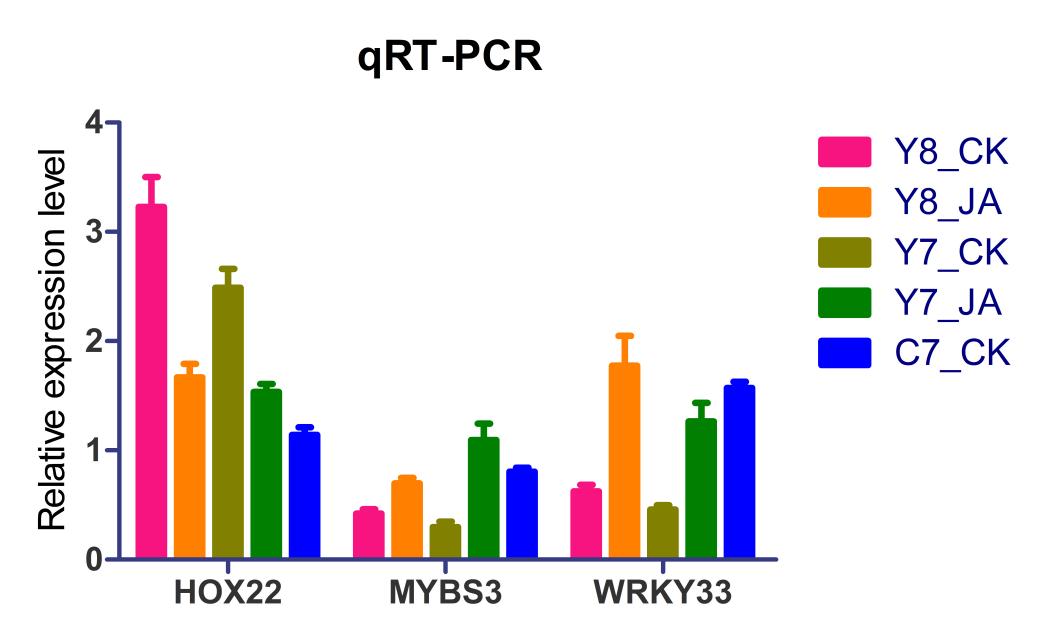


**Figure S3.** Expression levels of *HOX22*, *MYBS3*, and *WRKY33* in the maize spikelet.
